# Influence of indium-tin-oxide and emitting-layer thicknesses on light outcoupling of perovskite light-emitting diodes

**DOI:** 10.1186/s40580-019-0196-z

**Published:** 2019-08-08

**Authors:** Young-Jin Jung, Seong-Yong Cho, Jee-Won Jung, Sei-Yong Kim, Jeong-Hwan Lee

**Affiliations:** 1 Department of Materials Science and Engineering, Inha University, 100 Inha-ro, Michuhol-gu, Incheon, 22212 Republic of Korea; 2 LG Chem. Research Park, LG Chem. Co., Ltd, 188 Munji-ro, Yuseong-gu, Daejeon, 34122 Republic of Korea

**Keywords:** Outcoupling, Perovskite light-emitting diode, ITO thickness, Refractive index

## Abstract

Metal halide perovskite light-emitting diodes (PeLEDs) are emerging as a promising candidate for next-generation optoelectronic devices. The efficiency of PeLEDs has developed explosively in a short time, but their overall efficiency is still low. This is strongly related to the high refractive indexes of indium-tin-oxide (ITO) and perovskite emitting layers. Various outcoupling strategies are being introduced to outcouple the light trapped inside the layers. However, the proposed methods have experimental challenges that need to be overcome for application to large-area electronics. Based on optical simulations, we demonstrate that the thicknesses of the ITO and perovskite layers are key parameters to improve the outcoupling efficiency of PeLEDs. In addition, the optical energy losses of PeLEDs can be reduced significantly by properly adjusting the thicknesses of the two layers. This leads to outstanding optical performance with a maximum EQE greater than 20% without using any other external outcoupling strategies.

Metal halide perovskite light-emitting diodes (PeLEDs) are emerging as a promising option for next-generation flexible displays and lighting applications due to their outstanding optoelectrical characteristics, such as high color purity, easy spectrum tunability, and high brightness. These optoelectrical properties have been achieved by engineering the elements, structure, growth, and grain size of perovskite emitting-materials [1–15]. The developments in PeLEDs have given rise to improved quantum confinement, which has reduced the full-width half maximum of the emission spectrum to less than 20 nm. A high luminescence yield near unity has also been achieved as well as a tunable band-gap that can fully cover the visible spectral region [5, 10, 16]. Therefore, PeLEDs could be desirable candidates for achieving more vivid and clearer images compared to current LEDs, especially due to their high color purity and high brightness.

In the past several years, numerous strategies have been introduced to realize the full potential of PeLEDs. As a result, external quantum efficiency (EQE) as high as 20% has been achieved in PeLEDs in contrast to the first PeLED in 2014, which had less than 1% efficiency [3, 17–19]. However, the overall EQE of PeLEDs is still low since more than 70% of the photons generated are dissipated within the device [20–22]. The reason is strongly linked to the mismatch of the refractive index (*n*), between the perovskite emitting layer (EML, typically in the range of 2.0–2.3) and the other transporting layers (typically 1.6–1.8) in the multi-layered device. This discrepancy induces internal light reflections at the interface of the layers that restrict the outcoupling of PeLEDs. Therefore, improving the light outcoupling is an essential challenge to overcome [18, 19, 22–25].

Most of the strategies recently introduced for PeLEDs are focused on extracting photons from the device that are trapped in waveguide or substrate modes. For example, a submicrometer-scale structured perovskite EML gives photons a chance to change their propagation direction as soon as they escape the layer. Using the internal structure of the EML, Cao et al. reduced the waveguide optical losses and achieved a maximum EQE as high as 20% for the first time [19]. Another strategy is to extract photons from the substrate by using additional structures inside it [22, 24]. Desirable nanostructures with a proper difference in *n* can effectively help a large portion of confined photons to be outcoupled to the air, resulting in considerable improvements in EQE. However, there are additional experimental challenges in making nanostructures in an EML or substrate for large areas and reproducibility. A simple method is necessary to improve the outcoupling efficiency of PeLEDs for mass production.

Zhao et al. recently suggested a method to improve the EQEs of PeLEDs by adjusting only the thickness of the perovskite EML [25]. Using a simple strategy, they demonstrated a highly efficient PeLED with EQE greater than 17% without using any other outcoupling structures. The result suggests that adjusting the thicknesses of the layers is crucial and could lead to a simple technique to enhance the optical performances of PeLEDs.

In this study, we demonstrate that the thicknesses of the indium tin oxide (ITO) and perovskite layers are key parameters to improve the outcoupling efficiency of PeLEDs based on optical simulations. The ITO and perovskite layers have relatively high *n* values compared to the other layers, so photons are easily trapped in them. The results show that optimal thicknesses of the ITO and perovskite layers are 35 nm and 200 nm to reduce the optical losses via the waveguide and substrate modes. At these thicknesses, a maximum EQE greater than 20% can be realized without additional outcoupling structures.

Figure 1a shows the device structure of the PeLED and the molecular structure of the perovskite and transporting layers. A highly efficient red/near-infrared-emitting PeLED from the previous reports was used to investigate the effects of the thicknesses [3, 22, 25]. The PeLED is composed of glass (0.7 mm)/ITO (x nm)/poly(bis-4-butylphenyl-*N*,*N*-bisphenyl)benzidine (poly-TPD, 25 nm)/red-emitting perovskite layer (y nm)/2,2′,2″-(1,3,5-Benzinetriyl)-tris(1-phenyl-1-*H*-benzimidazole) (TPBi, 60 nm)/LiF (1 nm)/Al (100 nm). The ITO is a transparent conductive oxide layer, the poly-TPD is a hole transporting layer (HTL), the TPBi is an electron transporting layer (ETL), the LiF is an electron injecting layer, and the Al is a cathode.

**Fig. 1:**
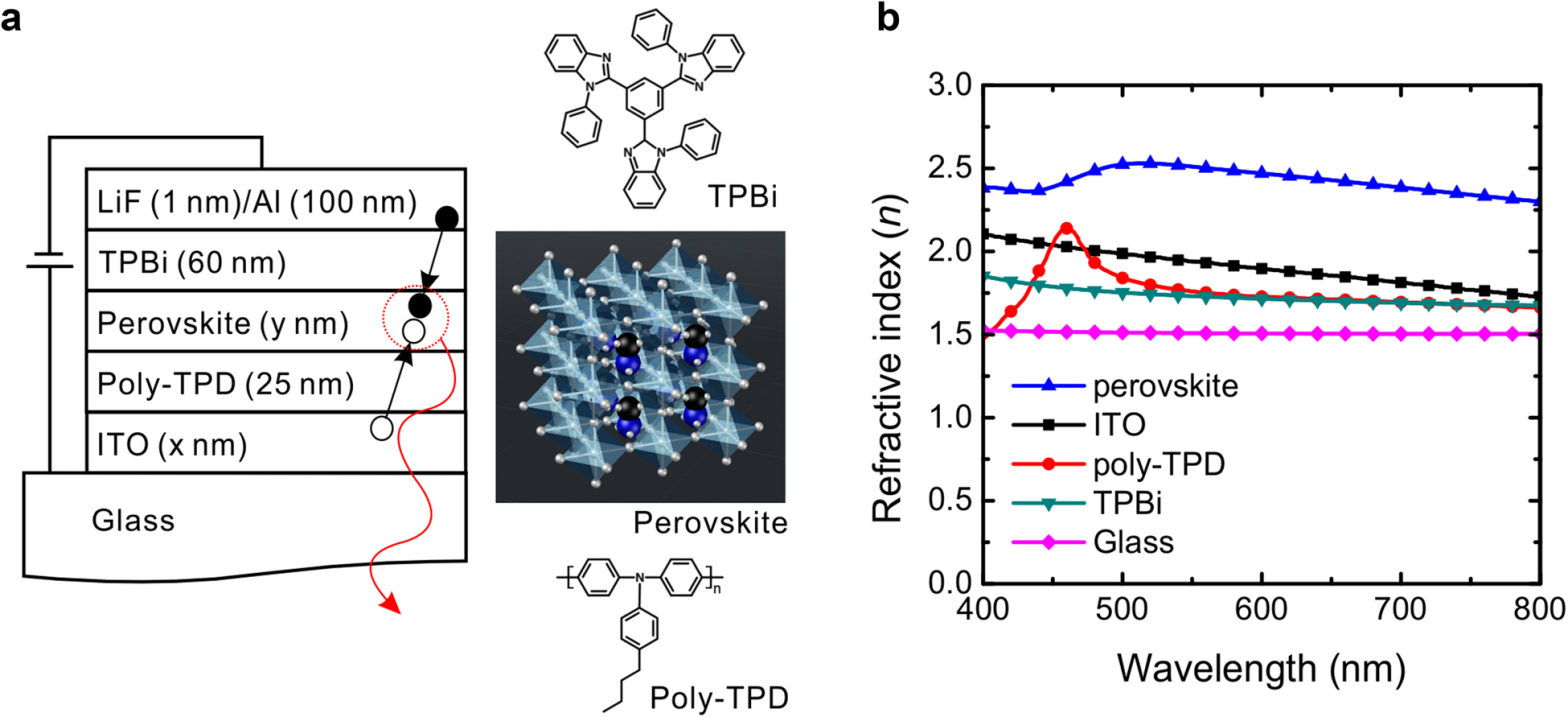
**a** Structure of the perovskite LED with a multi-layered structure for optical simulations. The molecular structures of the hole-transporting layer (poly-TPD), electron-transporting layer (TPBi), and emitting layer (perovskite) are included. **b** Refractive indexes of the layers in the perovskite LED as a function of wavelength

When external bias is applied to the PeLED, holes are injected from the ITO to the poly-TPD, and electrons are from the Al to the TPBi. Then, they move to the perovskite EML through the transporting layers. In the EML, they finally recombine and are converted to photons. The generated photons are outcoupled to the air through the multi-layered structure of the PeLED and they contribute to increasing the EQE of the device. During the process, most photons are annihilated within the device due to internal reflection, especially at the interfaces between different layers. More than 70% of the generated photons are typically trapped inside PeLEDs due to the internal reflection [20–22].

These optical energy losses are related to the *n* values of the layers in the device. The *n* mismatch between layers results in a strong internal reflection at the interface and a reduction of the EQE. Therefore, adjusting the *n* values of the layers is essential to boost the EQE of PeLEDs. Figure 1b shows the *n* values of the layers (ITO, poly-TPD, perovskite EML, and TPBi) of the PeLED shown in Fig. 1a. The ITO and perovskite layers have higher *n* values than the other layers. The large *n* mismatches at the interfaces between the perovskite and transporting layers (TPBi and poly-TPD) as well as between the ITO layer and glass substrate are likely to cause a large portion of the generated light to be reflected back at the interface. The light is confined within these layers when the incident angle of the light is less than the critical angle of the internal total reflection. The reflected light is proportional to *n* and the thickness of the layer in a multi-layered structure, so the architecture design is important for achieving high EQE.

Optical simulations were conducted to elucidate the effect of the thicknesses of ITO and perovskite layers based on a classical dipole model [26–28]. We assume that the emission zone is confined to an infinitely thin recombination zone and is located at the middle of the EML as sheet dipoles. We also assume that there is no electrical loss. The inputs to the simulation are the photoluminescence (PL) spectrum of the perovskite EML and the extinction coefficients of the layers, as shown in Fig. 2a, along with the *n* values in Fig. 1b. The PL quantum yield (PLQY) of the EML and the horizontal dipole ratio of the EML are 0.9 and 0.71 [3, 22].

**Fig. 2:**
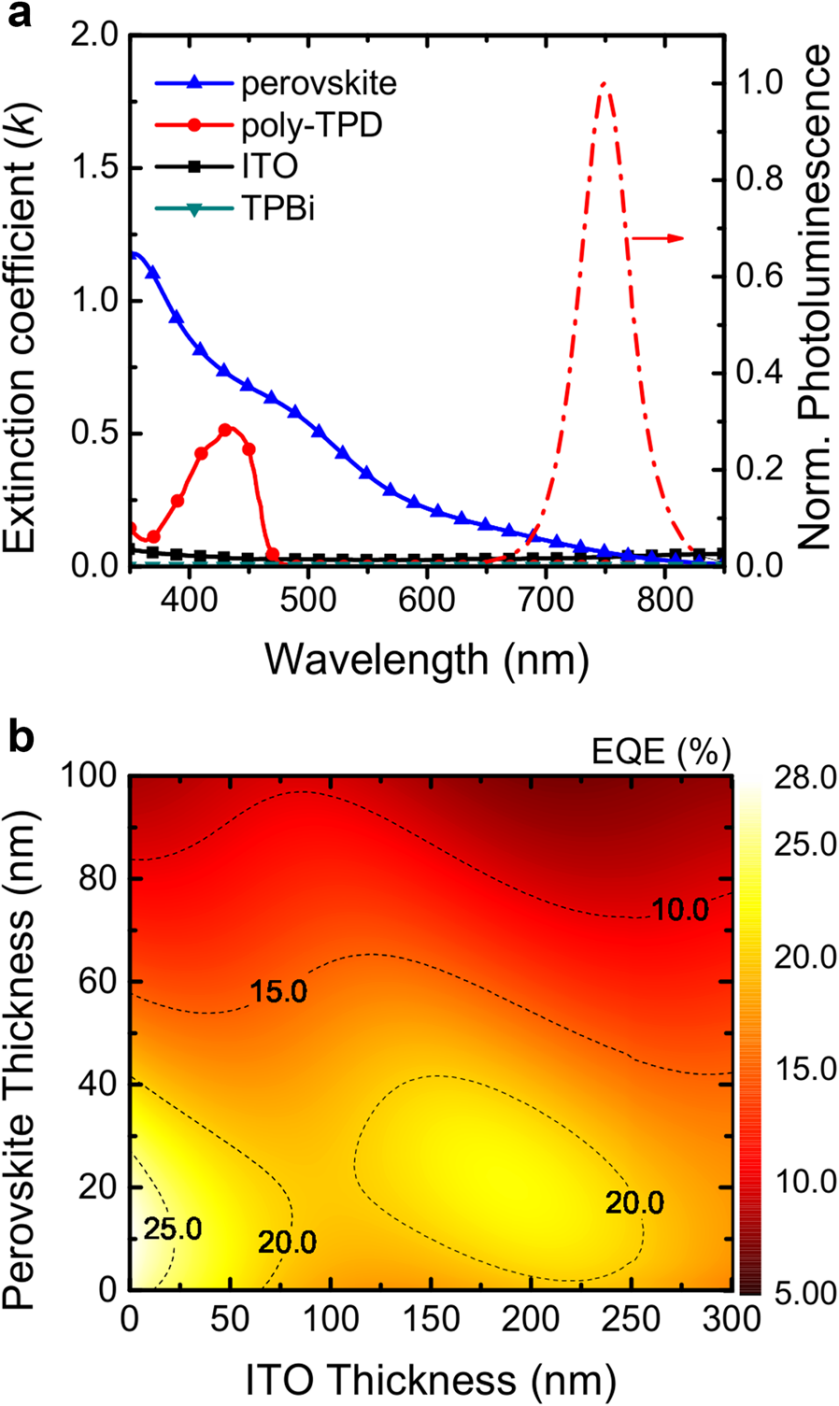
**a** Spectra of the extinction coefficients of perovskite, poly-TPD, ITO, and TPBi. The normalized photoluminescence spectrum of the perovskite layer is also included. **b** Contour plot of the maximum achievable EQE of the PeLED as a function of ITO and perovskite thicknesses

Figure 2a describes that the PL spectrum of the perovskite EML has a negligible spectral overlap with those of the extinction coefficients of the transporting layers. This indicates that the PeLED has no significant intrinsic optical energy losses from self-absorption and parasitic absorption. Figure 2b presents a contour plot of the maximum achievable EQE of the PeLED as a function of ITO and perovskite thicknesses. The EQE map has local maxima at an EML thickness of 10 nm and an ITO thickness of 0 nm, as well as at an EML thickness of 30 nm and an ITO thickness of 200 nm. Theses thicknesses correspond to the antinode positions in the cavity structure of the PeLED.

The EQE of the best PeLED reached more than 25% when the thicknesses of the ITO and perovskite layers were both less than 20 nm. However, the formation of thin layers is challenging for reproducibility. Moreover, it is hard to guarantee stable electrical conductivity with ITO electrodes that are less than 20 nm thick. Therefore, the best options are actually a 200-nm-thick ITO layer and a 30-nm-thick perovskite EML, which demonstrate a considerable EQE of 21%. These conditions offer a simple way to achieve highly efficient PeLEDs without any outcoupling structures.

At a fixed ITO thickness, the thickness of the perovskite EML plays an important role in improving the optical performance of the device. For instance, when the perovskite layer is 35 nm thick or 70 nm thick with a fixed ITO thickness of 200 nm, the EQE decreases significantly by almost half from 20 to 11%. The reason is the different mechanisms of optical energy loss, as shown in Fig. 3a, b. These figures present the optical power distributions for different ITO thicknesses with fixed thicknesses of the perovskite EML of 70 nm and 35 nm. The two cases show different power distribution behaviors. In the case of the 70-nm-thick perovskite EML (Fig. 3a), the main optical energy loss occurs via the waveguide mode (46.5%), followed by substrate mode (21.4%), absorption loss (10.1%), and surface plasmon polariton (SPP) loss (0.9%). The non-radiative loss is due to the PLQY of 0.9 from the EML. The result indicates that more than 45% of the generated light is confined inside the perovskite thick layer due to the high *n* of the EML. Only 11.2% of the optical energy is outcoupled to the air.

**Fig. 3:**
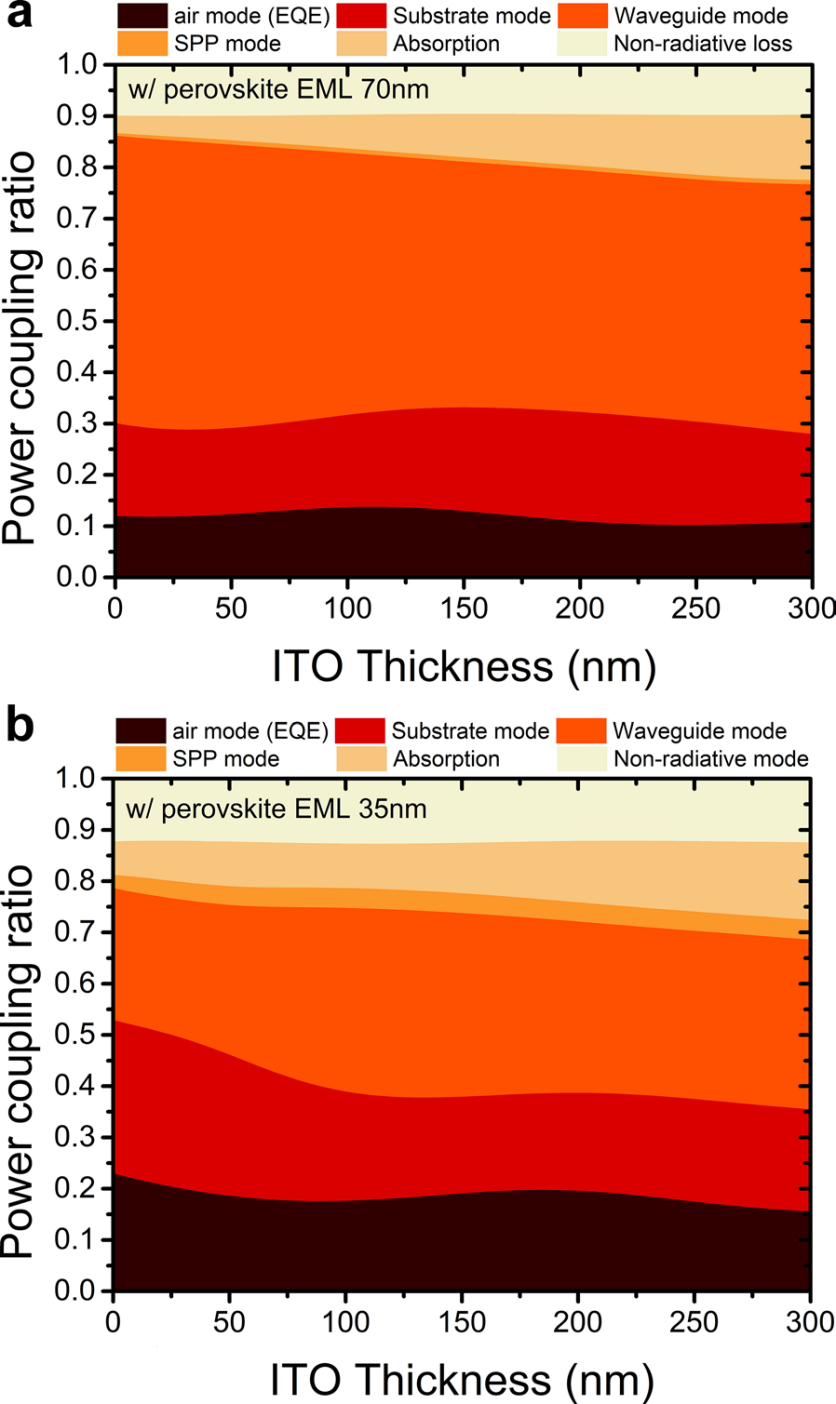
Optical power distributions for different ITO thickness with fixed thicknesses of the perovskite EML of **a** 70 nm and of **b** 35 nm

When the thickness of the perovskite layer is reduced to 35 nm, a certain portion of the confined light has another chance to be outcoupled. As a result, with the 70-nm-thick perovskite EML, there is less energy loss via the waveguide mode (31.4%) and more light, which contribute to the EQE (20.4%), as shown in Fig. 3b. The results clearly show that the EQEs of PeLEDs are strongly dependent on the thicknesses of the perovskite layer and ITO layer. Furthermore, the outcoupling efficiency of PeLEDs can be doubled by selecting proper thicknesses for the ITO and perovskite.

For further optimization, additional optical simulations were performed using different thicknesses of the transporting layers with fixed thicknesses of the ITO and EML at 200 nm and 35 nm. Figure 4 presents a contour plot of the maximum achievable EQE as a function of the thicknesses. The results indicate that the efficiency of the PeLED is more sensitive to the thickness of ETL than that of HTL due to the interference effect between the light emitted from the EML and the light reflected by the Al cathode. The EML is closer to the Al cathode than the HTL, so it plays an important role in forming constructive interference for the light in the cavity structure. At a fixed HTL thickness of 25 nm, the EQE varies widely with the ETL thickness from 5 to 20%. However, the HTL thickness has little impact on the EQE when the ETL thickness is fixed. The best PeLED achieved a high EQE of more than 20% when the thickness of the HTL was 30 nm and that of the ETL was 50 nm.

**Fig. 4:**
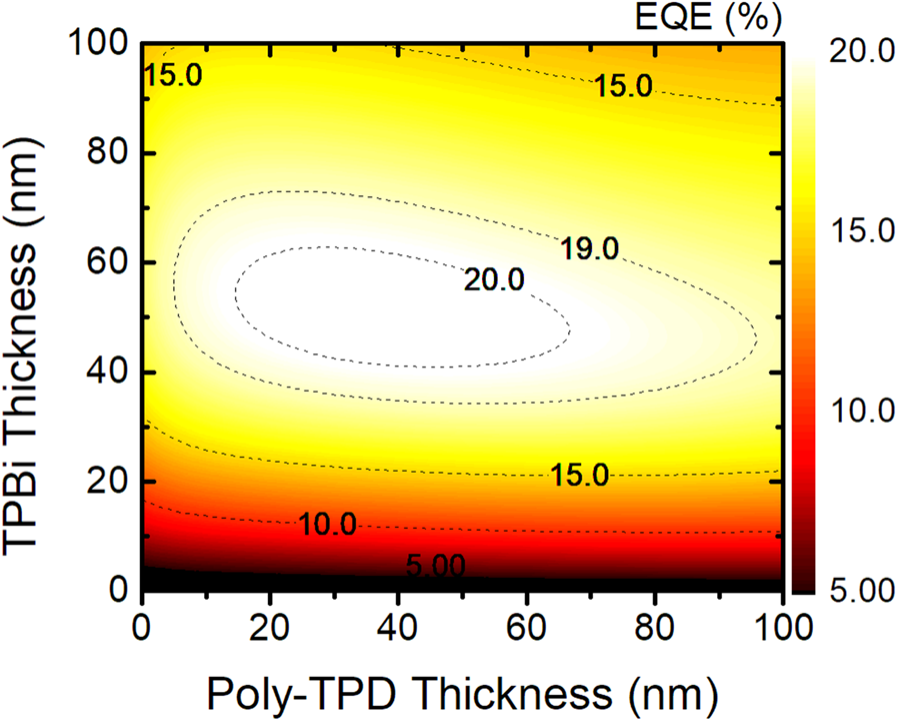
Contour plot of the maximum achievable EQE of the PeLED as a function of the thicknesses of the transporting layers, poly-TPD, and TPBi

In conclusion, we have proposed a simple strategy to enhance the efficiency of PeLEDs. The main approach is to compensate for the high *n* values of the ITO and perovskite emitting layers, which are one of the main bottlenecks in the optical performance of PeLEDs. The high *n* values result in the light being confined within the layers, leading to significantly lower efficiency. Based on systematic optical simulations, we investigated how to efficiently outcouple the confined light to the air and found that the EQEs of PeLEDs are strongly dependent on the thicknesses of the ITO and perovskite EML. The results indicated that a combination of a 30-nm-thick perovskite layer and a 200-nm-thick ITO layer reduces the optical energy losses via the substrate and waveguide modes. Therefore, outstanding optical performance could be realized with EQE greater than 20% without any other outcoupling structures.

## Data Availability

The datasets used in this study are available from the corresponding author upon reasonable request.

## Abbreviations

PeLEDs: perovskite light-emitting diodes
EQE: external quantum efficiency
*n*: refractive index
EML: emitting layer
ITO: indium tin oxide
poly-TPD: poly(bis-4-butylphenyl-*N*,*N*-bisphenyl)benzidine
TPBi: 2,2′,2″-(1,3,5-benzinetriyl)-tris(1-phenyl-1-*H*-benzimidazole)
LiF: lithium fluoride
Al: aluminum
HTL: hole-transporting layer
ETL: electron-transporting layer
PL: photoluminescence
PLQY: photoluminescence quantum yield
SPP: surface plasmon polariton
